# Providing dermatologic care in Botswana

**Published:** 2010-12-09

**Authors:** Aileen Yenting Chang, Carrie Lynn Kovarik

**Affiliations:** 1 Department of Dermatology, University of Pennsylvania School of Medicine, Philadelphia, PA, USA; 2 Division of Infectious Diseases, Department of Medicine, University of Pennsylvania School of Medicine, Philadelphia, PA, USA

**Keywords:** Clinical dermatology, economic issues, social issues

## Abstract

**Abstract:**

Dermatologists practicing in countries with limited resources are faced with multiple challenges, including medication unavailability/affordability, infrequency of clinics, and illiteracy. We share our experiences providing care in dermatology clinics throughout Botswana. We discuss the challenges we faced and several of the solutions we implemented. Lessons learned are applicable to similar resource-limited settings in Africa.

## To the editors of Pamj

We would like to share our recent experiences providing care in dermatology clinics throughout Botswana. We depict challenges faced and solutions implemented.

In the capital city of Gaborone, the dermatology clinic at a tertiary public hospital is staffed by a foreign dermatologist, and one to two American or Canadian senior dermatology resident, who rotate for four to six weeks. One of the biggest challenges faced was utilizing the government formulary to its fullest potential. We learned to be flexible and optimize use of the drugs available. For example, dapsone is on the formulary for treating leprosy, but we also used it for neutrophilic dermatoses and several autoimmune blistering diseases. Additionally, for over a month, we experienced a hospital-wide shortage of doxycycline. This was the best formulary medication for treating common dermatologic problems, including bacterial skin infections and acne vulgaris. Fortunately, doxycycline is an inexpensive medication (1.5Pula/0.22USD per tablet), and all of our outpatients who were not treated effectively with formulary topical medications were willing to pay out-of-pocket at local pharmacies. We found that providing the expected weekly/monthly cost was effective in demonstrating the affordability of the medication. For inpatient consults, we turned to erythromycin for infection prophylaxis in patients with skin compromise. Another challenge was the expense of sunscreen. Patients with photosensitive conditions, such as cutaneous lupus and oculocutaneous albinism, were repeatedly advised to wear sunscreen, yet virtually all were non-compliant. Sunscreen is not on the formulary and is very expensive to purchase locally (96Pula/14USD for 100mL). Recommendations for more affordable interventions, such as hats and umbrellas, were embraced by these patients, especially after they understood the relationship of the sun to their skin disorder.

To educate patients, we engaged in verbal teaching and provided information pamphlets for common conditions. For illiterate patients, nurses read the information in the local language. In district clinics, located in villages outside of Gaborone, clinic is held once a month and staffed by the rotating residents. In these remote locations, we faced similar challenges as in Gaborone, in addition to others. Since dermatology clinic was held infrequently, our patients’ primary lesion had often resolved by the time they were seen in clinic. Our diagnosis became based on the patient’s history and the distribution of post-inflammatory lesions (i.e. hyper/hypopigmentation). When this approach was insufficient, we instructed patients to take a picture of the lesion and bring that photograph to next month’s clinic. This was a feasible solution for most patients, as many had a camera on their cellular phone.

A minority of patients seen at district clinics was illiterate. This was particularly a challenge when we needed patients to purchase over-the-counter products. For example, Vaseline® petroleum jelly, unavailable at the clinic’s pharmacy, is frequently used for treating xerosis and for compounding topical medications. We found that showing a photograph of the product was the most effective way to explain which product to purchase.

Lastly, detailed documentation was invaluable, particularly in our situation where patients usually see different physicians at the initial visit and subsequent follow-up visits. Skin lesions evolve over time, with or without treatment, and the assessment of a lesion depends significantly on physical exam findings. Thus, it was essential for each physician to provide a detailed physical description and differential diagnosis at each visit.

Dermatologists practicing in countries with limited resources are faced with multiple challenges, including medication unavailability/affordability, infrequency of clinics, and illiteracy. Some challenges may require changes to infrastructure, whereas others have feasible solutions that clinicians can implement with relative ease.

## Competing interests

The authors declare no conflicts of interest.

